# Pancreatic Cancer Stem Cells Co-Expressing SOX2, OCT4, and TERT^high^ Represent an Aggressive Subpopulation

**DOI:** 10.3390/cells15020129

**Published:** 2026-01-11

**Authors:** Erika Curiel-Gomez, Damaris P. Romero-Rodriguez, Mauricio Rodriguez-Dorantes, Vilma Maldonado, Jorge Melendez-Zajgla

**Affiliations:** 1 Posgrado en Ciencias Biológicas, Unidad de Posgrado, Edificio D, 1° Piso, Circuito de Posgrados, Ciudad Universitaria, Coyoacán, Mexico City C.P. 04510, Mexico; erika.curiel.g@gmail.com; 2 Laboratorio de Genómica Funcional del Cáncer, Instituto Nacional de Medicina Genómica, Periférico Sur No. 4809, Col Arenal Tepepan, Tlalpan, Mexico City C.P. 14610, Mexico; 3 Laboratorio Nacional Conahcyt de Investigación y Diagnóstico por Inmunocitofluorometría (LANCIDI), Instituto Nacional de Enfermedades Respiratorias “Ismael Cosío Villegas”, Calzada de Tlalpan 4502, Col. Belisario Domínguez Secc. 16, Tlalpan, Mexico City C.P. 14080, Mexico; lancidi@iner.gob.mx; 4 Laboratorio de Oncogenómica, Instituto Nacional de Medicina Genómica, Periférico Sur No. 4809, Col Arenal Tepepan, Tlalpan, Mexico City C.P. 14610, Mexico; mrodriguez@inmegen.gob.mx; 5 Laboratorio de Epigenética, Instituto Nacional de Medicina Genómica, Periférico Sur No. 4809, Col Arenal Tepepan, Tlalpan, Mexico City C.P. 14610, Mexico; vmaldonado@inmegen.gob.mx

**Keywords:** pancreatic cancer, telomerase, SOX2-OCT4, cancer stem cells

## Abstract

The aggressiveness of pancreatic ductal adenocarcinoma (PDAC) has been linked to cancer stem cells (CSCs) and telomerase activity; however, the mechanism underlying this association remains unclear. In this study, we engineered dual transcriptional reporters (SORE6-GFP and TERT-BFP) to isolate SOX2^+^OCT4^+^TERT^high^ subpopulations from AsPC-1 and BxPC-3 cells. We combined Fluorescence-Activated Cell Sorting with functional assays, RNA-seq, and network analysis. Clinically, tumors co-expressing high SOX2/OCT4/TERT levels were associated with reduced overall survival, whereas single-gene elevations were not prognostic. We identified a minority SOX2^+^OCT4^+^TERT^high^ fraction (~9%) enriched for pluripotency transcripts (*SOX2*, OCT4, NANOG, and ALDH1A1), which exhibited the highest proliferative, migratory, and invasive capacities. Transcriptomic profiling of SOX2^+^OCT4^+^TERT^high^ cells showed enrichment of KRAS, telomere maintenance, epithelial–mesenchymal transition, and developmental pathways (WNT and Hedgehog). Connectivity profiling highlighted actionable vulnerabilities, including NF-κB, WNT, and telomerase inhibition pathways. Together, these data define an aggressive telomerase-engaged, pluripotency-driven CSC-like state in PDAC and suggest testable therapeutic strategies that target TERT^high^ dependencies.

## 1. Introduction

Pancreatic ductal adenocarcinoma (PDAC) is the most frequent and deadliest form of pancreatic cancer, with a 5-year survival rate of approximately 12% [1]. Alarmingly, PDAC is projected to become the second leading cause of cancer-related deaths by 2040 [2]. Despite therapeutic advances, numerous factors drive PDAC lethality, including the inability to detect the disease until late in progression, often after distant metastasis and marked resistance to chemotherapy and radiotherapy [3]. Two combination regimens have emerged as the first-line therapy in metastatic pancreatic ductal adenocarcinoma, FOLFIRINOX and the combination of gemcitabine and nab-paclitaxel. However, significant toxicity and resistance constrain their benefits [4,5]. There is an urgent need to identify efficient therapeutic strategies for patients with PDAC.

PDAC tumors are characterized by pronounced cellular heterogeneity, including cancer stem cell (CSC) populations, which are thought to sustain progression, chemoresistance, and metastasis [6,7]. Pancreatic CSCs are distinguished by their ability to self-renew and generate differentiated cells. Recent studies suggest that CSC properties can be gained and lost depending on the microenvironment, indicating that CSCs are not stable but rather a highly plastic cell population [8]. Several markers, including CD133, CD24, CD44, *NANOG*, *SOX2*, and *POU5F1* (OCT4), have been proposed for the identification of CSCs in PDAC, indicating high heterogeneity within this population [9,10,11]. *SOX2* is aberrantly expressed in aggressive PDAC cells and contributes to cell proliferation and stemness/dedifferentiation by regulating a set of genes controlling G1/S transition and epithelial-to-mesenchymal transition (EMT) phenotype. OCT4 is overexpressed and has been implicated in stemness, carcinogenesis, metastasis, drug resistance, and epithelial–mesenchymal transition (EMT) [10,11].

Telomerase reverse transcriptase (TERT), an RNA-dependent DNA polymerase, synthesizes telomeric DNA and adds it to the linear chromosome ends to maintain telomere length [12]. Beyond its canonical role, TERT has been implicated in non-telomeric functions that influence survival signaling, metastasis, and stem cell self-renewal [13]. Mechanistically, TERT acts as a transcriptional cofactor that potentiates two oncogenic axes. TERT associates with BRG1 to activate the promoters of WNT/β-catenin target genes, such as Myc and CyclinD1 [14]. TERT also binds to the NF-κB p65 subunit and is recruited to NF-κB-responsive promoters, enhancing the expression of genes linked to tumor progression [15]. Telomerase expression is a near-universal feature of cancer [16]. In the pancreas, telomerase is upregulated in invasive PDAC, and polymorphisms in the TERT locus are associated with an increased risk of PDAC [17]. Additionally, TERT^high^ cells have been proposed to tolerate and maintain oncogenic KRAS, promoting cell expansion and creating fields of aberrant cells that seed tumorigenesis [18]. Nevertheless, whether TERT is expressed in pancreatic CSCs and how TERT-positive cells contribute to progression remain unresolved.

Here, we address this gap using a dual-reporter strategy, SOX2/OCT4-response element-driven Green Fluorescent Protein (GFP) and TERT-promoter-driven Blue Fluorescent Protein (BFP), to isolate SOX2^+^OCT4^+^TERT^high^ and SOX2^+^OCT4^+^TERT^low^ subpopulations from pancreatic cancer cell lines and investigate their molecular, functional, and phenotypic properties. We found that the subpopulation of SOX2^+^OCT4^+^TERT^high^ cells exhibited enhanced proliferation, migration, and invasion, along with a transcriptomic program enriched for telomere maintenance, pluripotency, EMT, and developmental pathways. Causal network analysis supported the coordinated activation of these signaling axes. Finally, we identified compounds specific to the selected molecular targets and mechanisms that may eventually lead to novel treatments for aggressive TERT^high^ cells.

## 2. Materials and Methods

### 2.1. Bioinformatic Analysis

Pearson’s correlation between the cancer stemness index and TERT expression was calculated using the stemness index (mRNAsi) for *The Cancer Genome Atlas* (TCGA) downloaded from a pan-cancer study [19], and TERT mRNA expression levels in pancreatic cancer were obtained from the TCGA PanCan Atlas.

To evaluate the expression levels of *SOX2*, *POU5F1* (OCT4), and *TERT*, RNA-seq data from three publicly available sources were used: pancreatic cancer tissues from TCGA PanCan Atlas https://gdc.cancer.gov/node/905/ (accessed on 3 May 2025), healthy pancreatic tissue from the GTEx portal https://www.gtexportal.org/home/ (accessed on 3 May 2025), and the pancreatic cancer cell lines AsPC-1 and BxPC-3 from the *Cancer Cell Line Encyclopedia* https://sites.broadinstitute.org/ccle (accessed on 3 May 2025).

The prognostic impact of *SOX2*, OCT4 and *TERT* expression on overall survival (OS) and relapse-free survival (RFS) in pancreatic ductal adenocarcinoma patients was evaluated using the Kaplan–Meier Plotter https://kmplot.com/analysis/index.php?p=home (accessed on 9 May 2025). Patients were stratified into high- and low-expression groups based on *SOX2*, OCT4, TERT, and the combined SOX2/OCT4/TERT signature.

### 2.2. Cell Culture and Reagents

AsPC-1, BxPC-3 pancreatic cancer, and HEK-293T cells were obtained from the American Type Culture Collection (ATCC, Manassas, VA, USA). All cell lines were authenticated by the ATCC. Cells were cultured according to the ATCC recommendations. AsPC-1 and BxPC-3 cells were cultured in RPMI 1640 medium (ATCC 30-2001, Manassas, VA, USA) supplemented with 10% fetal bovine serum (FBS) (Gibco, Cat. No. 16000044; Thermo Fisher Scientific, Waltham, MA, USA) and 1% penicillin–streptomycin (10,000 U/mL; Cat. No. 15140122. Thermo Fisher Scientific, Waltham, MA, USA). HEK-293T cells were maintained in Dulbecco’s Modified Eagle Medium (DMEM; ATCC 30-2002, Manassas, VA, USA) supplemented with 10% FBS and 1% penicillin–streptomycin for lentiviral production. The cell lines were cultured at 37 °C in a 5% CO_2_ humidified atmosphere. When the cells reached approximately 80% confluency, they were trypsinized, harvested, washed, and used for experiments or passaged for further expansion.

### 2.3. Generation of Lentiviral Reporter Constructs

To mark pluripotency factor activity, the SORE6-GFP lentiviral reporter (six concatenated SOX2/OCT4 response elements driving GFP) was used [20]. SORE6-GFP lentiviral reporters were kindly provided by Dra. Lalage M. Wakefield (National Cancer Institute (NCI), Bethesda, MD, USA). To mark telomerase transcription, a TERTpromoter driving the BFP lentiviral reporter was generated. The blue fluorescent protein (BFP) sequence was synthesized (Integrated DNA Technologies, IDT, Coralville, IA, USA) and amplified using ClonaAmp HiFi premix (Takara Bio USA, Mountain View, CA, USA). PCR product purification was performed using the QIAquick PCR purification Kit (QIAGEN, Germantown, MD, USA). BFP was cloned into the lentiviral reporter vector pL-mP (Addgene_81225, Watertown, MA, USA), replacing the green fluorescent protein (GFP) sequence with the BFP. The human telomerase promoter region, which originates from the HR-TERT-promoter vector (Addgene_71398, Watertown, MA, USA), was obtained by PCR using ClonaAmp HiFi premix (Takara Bio, Mountain View, CA, USA). PCR product purification was performed using the QIAquick PCR purification Kit (QIAGEN, Germantown, MD, USA). The TERT promoter was cloned into the pL-mP-BFP vector using an In-Fusion cloning kit (Takara Bio, Mountain View, CA, USA). In contrast, to generate the control vector CMV-BFP, the BFP sequence was cloned into the vector pcDNA 3.1 (Invitrogen Thermo Fisher Scientific, Waltham, MA, USA, Cat. No. V790-20). E. coli One Shot Stbl3 electrocompetent cells (Thermo Fisher Scientific, Rockford, IL, USA, Cat. No. C737303) were used for plasmid amplification. The QIAprep Spin Miniprep Kit (QIAGEN, Germantown, MD, USA) and HiSpeed Plasmid Midi Kit were used for all plasmid purification (QIAGEN, Germantown, MD, USA, Cat. No. 12643). All inserts and junctions were verified by Sanger sequencing.

### 2.4. Generation of Lentiviruses and Infections

To identify SOX2^+^OCT4^+^TERT^high^ cells, TERT-BFP and SORE6-GFP lentiviral reporter vectors were used. Lentiviral particles were produced by co-transfecting HEK-293T cells with a lentiviral packaging system (pCMV-VSV-G_8454 and psPAX_12260, Addgene (Watertown, MA, USA)) and transfer plasmid (Table A1, Appendix B) using Lipofectamine^TM^ 2000 (Invitrogen, Waltham, MA, USA) reagent according to the manufacturer’s protocol. Before transfection, plasmid DNA was purified using an endotoxin-free Maxiprep kit (Qiagen Cat. No. 12362, Germantown, MD, USA to ensure optimal transfection efficiency and to prevent cytotoxicity. Viral supernatants were harvested after 48 and 72 h post-transfection and filtered through a 0.45 μm pore size membrane (Millipore Thermo Fisher Scientific, Waltham, MA, USA). The viral pellet was stored at −80 °C in single-use aliquots to avoid repeated freeze-thaw cycles. To generate double-reporter cell lines, human AsPC-1 and BxPC-3 pancreatic tumor cells were infected with TERT-BFP viral supernatants supplemented with 8 μg/mL polybrene (TR-1003-G, Millipore Sigma, Burlington, MA, USA). Subsequently, BFP^+^ cells were isolated by FACS, returned to culture, and transduced with the second reporter (SORE-6) supplemented with 8 μg/mL polybrene and selected with 2–10 μg/mL puromycin (P9620, Sigma-Aldrich, Millipore Sigma, Burlington, MA, USA) for 15 days to select transfected cells. To generate GFP^+^, BFP^−^, and GFP^−^ control cell lines, cells were infected with CMV-GFP (pLJM1-EGFP, Addgene_19319 (Watertown, MA, USA)), mP-BFP, or minCMVp-GFP, respectively, supplemented with 8 μg/mL polybrene, and selected with 2–10 μg/mL puromycin or FACS. The transduction efficiency was typically >80%. 

To generate BFP^+^ control cells, the cells were transfected with the CMV-BFP vector using Lipofectamine 2000^TM^ (Invitrogen, Thermo Fisher Scientific, Waltham, MA, USA) according to the manufacturer’s protocol. After 24 h, transduced cells were selected by the addition of 200 μg/mL of G418 (A1720; Sigma-Aldrich, Millipore Sigma, Burlington, MA, USA) for 30 days to select transfected cells.

### 2.5. Flow Cytometry and Fluorescence-Activated Cell Sorting (FACS)

Subconfluent double-reporter AsPC-1 and BxPC-3 cell lines were collected by trypsinization, washed with PBS, and resuspended in buffer (PBS with FBS 1%). Flow cytometry and FACS were performed using BD FACSAria II (Becton Dickinson, Franklin Lakes, NJ, USA) for GFP and BFP expression. The sorted cells were harvested and grown under standard culture conditions for 24 h before any further procedures. Cells transduced with minCMVp-GFP or mP-BFP lentivirus were used as matched negative controls for gating. Cells generated with the CMV-GFP or CMV-BFP vectors were used as compensation controls. Sorted fractions were defined as SOX2^+^OCT4^+^TERT^high^ (GFP^high^BFP^high^), SOX2^+^OCT4^+^TERT^low^ (GFP^high^BFP^low^) and SOX2^−^OCT4^−^TERT^−^ (GFP^−^BFP^−^). Flow cytometry data were analyzed using the FlowJo version 10.8.1 software package (Tree Star, Ashland, OR, USA).

### 2.6. Analysis of Stemness Markers by Flow Cytometry

For comparative phenotyping, a total of 4 × 10^5^ sorted cells were stained with CD24-PE (clone ML5, 555428, BD Biosciences, San Jose, CA, USA) and EpCAM-APC (347200), or cells were stained with CD133-PE (clone AC133, 130-112-157, Miltenyi Biotec, Bergisch Gladbach, Germany) and EpCAM-APC (clone HEA125, 347200, BD Biosciences, San Jose, CA, USA) for 30 min on ice. The isotypes IgG2a-PE (clone 130-117-787, Miltenyi Biotec, Bergisch Gladbach, Germany), IgG1-PE (clone 130-092-212, Miltenyi Biotec, Bergisch Gladbach, Germany), and IgG1-APC (clone 130-092-214, Miltenyi Biotec, Bergisch Gladbach, Germany) were used as the staining control. Cells were analyzed using a BD FACSAria II instrument (Beckton Dickinson, Franklin Lakes, NJ, USA), and the data were analyzed using the FlowJo software package.

### 2.7. Fluorescence Microscopy

Sorted cell populations were grown on glass coverslips in a 6-well plate and fixed with 4% paraformaldehyde (Sigma-Aldrich, St. Louis, MO, USA) for 30 min. The cells were washed with PBS, and the slides were mounted in Everbrite mounting medium (Biotium Inc., Hayward, CA, USA) and stored at 4 °C. Images were acquired using a ZEISS-Axio Scope.A1 microscope (Carl-Zeiss, Oberkochen, Germany) and analyzed using ImageJ version 2.16.0 (National Institutes of Health, Bethesda, MD, USA) and Adobe Photoshop version 25.3.1 (Adobe Inc., San Jose, CA, USA).

### 2.8. RT-PCR and Quantitative Real-Time PCR (qPCR)

Total RNA was isolated from sorted cell populations using TRIzol reagent (Life Technologies, Thermo Fisher Scientific, Waltham, MA, USA) according to the manufacturer’s instructions. RNA concentration and purity were determined using a NanoDropTM2000c (Thermo Fisher Scientific, Waltham, MA, USA) spectrophotometer by measuring the A260/A280 ratio, and integrity was verified by 1% agarose gel electrophoresis. Two micrograms of RNA were reverse transcribed using the Maxima First Strand cDNA Synthesis Kit (Thermo Fisher Scientific, Rockford, IL, USA) with random hexamer primers (Invitrogen). Quantitative real-time PCR was conducted using the SYBR-select Master kit (Thermo Fisher Scientific, Rockford, IL, USA) and amplified on a QuantStudio 7 Real-Time PCR System (Applied Biosystems, Thermo Fisher Scientific, Waltham, MA, USA) using SYBR Green chemistry. Primer sequences provided in Table A2 Appendix B were designed in the Primer 3 plus software. The relative expression level of each gene was normalized to the peptidyl-prolyl cis-trans isomerase (*PPIA*) gene and calculated using the CT value based on the 2^−ΔΔCt^ method [21].

### 2.9. Cell Proliferation Assays

Cell proliferation was assessed by the MTS assay using the Cell Titer 96 Aqueous One Solution Cell Proliferation Assay Kit (G5430, Promega Corporation, Madison, WI, USA). Briefly, 5000 sorted cell populations in RPMI-1640 medium without phenol red (Sigma-Aldrich, MilliporeSigma, Burlington, MA, USA) and free of FBS were seeded in 96-well plates and incubated at 37 °C in 5% CO_2_. Cell proliferation was evaluated at 24, 48, 72, and 96 h. The cells were incubated with the MTS reagent for 3 h at 37 °C in a 5% CO_2_ humidified atmosphere. The light absorbance of each well was measured using a Beckman Coulter DTX 880 Multimode Detector Spectrophotometer at a wavelength of 450 nm. As a negative control, cells were incubated in the presence of Ara-C 2 mM (C1768, Sigma-Aldrich, MilliporeSigma, Burlington, MA, USA) to inhibit proliferation.

### 2.10. In Vitro Cell Migration Assays

Cell migration capacity was evaluated using wound-healing assays. Sorted cell populations were suspended in a culture medium at 200,000 cells/mL, and 70 μL cell suspensions were pipetted into each chamber of the cell culture insert (Ibidi^®^ 35 mm Culture Dishes containing Culture-Insert 2 Well systems. Cat. No. 80206; Ibidi GmbH, Gräfelfing, Germany). After the initial 24 h attachment period, the Culture-Insert 2 Well was gently removed using sterile forceps to create a defined cell-free gap (wound) of 500 μm, and the cells were incubated at 37 °C in a 5% CO_2_ humidified atmosphere. To ensure that wound closure reflected cell migration rather than proliferation, the medium was supplemented with 2 μM cytosine β-D-arabinofuranoside hydrochloride (C1768, Sigma-Aldrich, MilliporeSigma, Burlington, MA, USA) as a DNA synthesis inhibitor. Images were captured at 0 and 72 h at 10× magnification using an Eclipse TS100 microscope (Nikon, Kawasaki, Japan), and the cell-free space was determined using ImageJ software. Cell migration was represented as the percentage of wound closure.

### 2.11. In Vitro Cell Invasion Assays

The cell invasion capacity was evaluated using Boyden chambers. A 24-well Corning^®^ BioCoat™ Matrigel^®^ Invasion Chamber (354480, Corning Life Sciences, Corning, NY, USA) was used for the assay. The assay was performed according to the manufacturer’s instructions. Briefly, the Matrigel inserts were rehydrated using a medium free of SFB. After rehydration, 50,000 BxPC-3 or 100,000 AsPC-1 sorted cells were seeded in the upper chamber and cultured in serum-free medium. The lower chambers were loaded and filled with 600 μL medium containing 10% FBS as a chemoattractant or without FBS as a negative control. Cells were cultured at 37 °C in a 5% CO_2_ humidified atmosphere for 24 h. Inserts were then removed from the wells, and the cells on the upper surface of the Transwell membrane were removed. Migrating cells on the lower surface were rinsed with PBS, fixed with 70% ethanol, and stained with 0.1% crystal violet stain. Images were captured using an Eclipse TS100 microscope (Nikon, Kawasaki, Japan), and the migrating cells were quantified using ImageJ software.

### 2.12. Bulk RNA Sequencing

For RNA-seq analysis of SOX2^+^OCT4^+^TERT^low^ and SOX2^+^OCT4^+^TERT^high^ AsPC-1 cells, total RNA was isolated from sorted cell populations using TRIzol reagent (Life Technologies, Thermo Fisher Scientific, Waltham, MA, USA) according to the manufacturer’s instructions. RNA concentration and integrity were determined using a Qubit RNA Assay (Thermo Fisher Scientific, Waltham, MA, USA) and Bioanalyzer 2100/TapeStation (Santa Clara, CA, USA), respectively. Samples with an RNA integrity number of 10 were considered for transcriptome sequencing. Total RNA sequencing was performed using a NextSeq2000 (Illumina, San Diego, CA, USA) system with a paired-end 2 × 100 bp configuration, following the manufacturer’s standard protocol. Three biological replicates were used for analysis. RNA-seq processing and differential expression analyses were performed using the institute’s standardized workflows with minor modifications. Complete documentation of tools, parameters, and statistical criteria is publicly available online https://github.com/INMEGEN/Pipelines_Inmegen/tree/Principal (accessed on 30 May 2025). Clean reads were aligned to the GRCh 38 version of the human genome using the STAR algorithm and quantified using the FeatureCounts package. Differential expression between groups was determined using the DESeq2 algorithm. Genes with a fold change increment higher than 2 or less than −2, a *p*-value ≤ 0.05, and adjusted *p*-values (false discovery rate, FDR) ≤ 0.1 were further considered for subsequent analyses. To validate the RNA-seq data, qPCR was performed on differentially expressed genes. We selected differentially expressed genes with high read counts because the variance in those data is lower and the differences are more reliable. We also checked the expression values of these transcripts across replicates and chose genes with constant read counts between replicates.

### 2.13. Gene Set Enrichment Analysis (GSEA)

Data obtained from our RNA-seq was imported into the GSEA version 4.3.2 software downloaded from the following website: https://software.broadinstitute.org/gsea/index.jsp (accessed on 3 May 2025). Sets of genes related to different gene ontology processes served as reference genes to determine the biological processes enriched in our data. We only considered the gene set enrichment dataset with an FDR < 0.25.

### 2.14. Key Pathway Analysis

Ingenuity Pathway Analysis software (IPA-QIAGEN) version Q4 2025 was used to predict the regulatory networks and infer essential cellular pathways in SOX2^+^OCT4^+^TERT^high^ cells. This tool uses a list of differentially expressed genes obtained from RNA-seq.

### 2.15. Compounds Targeting Cells SOX2^+^OCT4^+^TERT^high^

To determine which target drugs might be useful against SOX2^+^OCT4^+^TERT^high^ cells, the list of differentially expressed genes obtained from our RNA-seq analysis was analyzed using the Connectivity Map https://clue.io/ (accessed on 5 August 2025) of the Broad Institute to predict compounds that can activate or inhibit gene expression signatures. To further investigate the mechanism of action (MoA) and drug targets, we performed a specific analysis using Connectivity Map tools https://clue.io/query (accessed on 10 August 2025). 

### 2.16. Statistical Analysis

Statistical analysis was performed using GraphPad Prism 7, and a 95% confidence interval was considered. A *t*-test or one- or two-way ANOVA with Tukey’s post hoc test was used to calculate statistical significance. All experiments were performed at least in triplicate.

## 3. Results

### 3.1. SOX2, OCT4, and TERT Are Expressed in Pancreatic Cancer, and Their Expression Affects the Survival Outcomes of Patients

**Figure 1:**
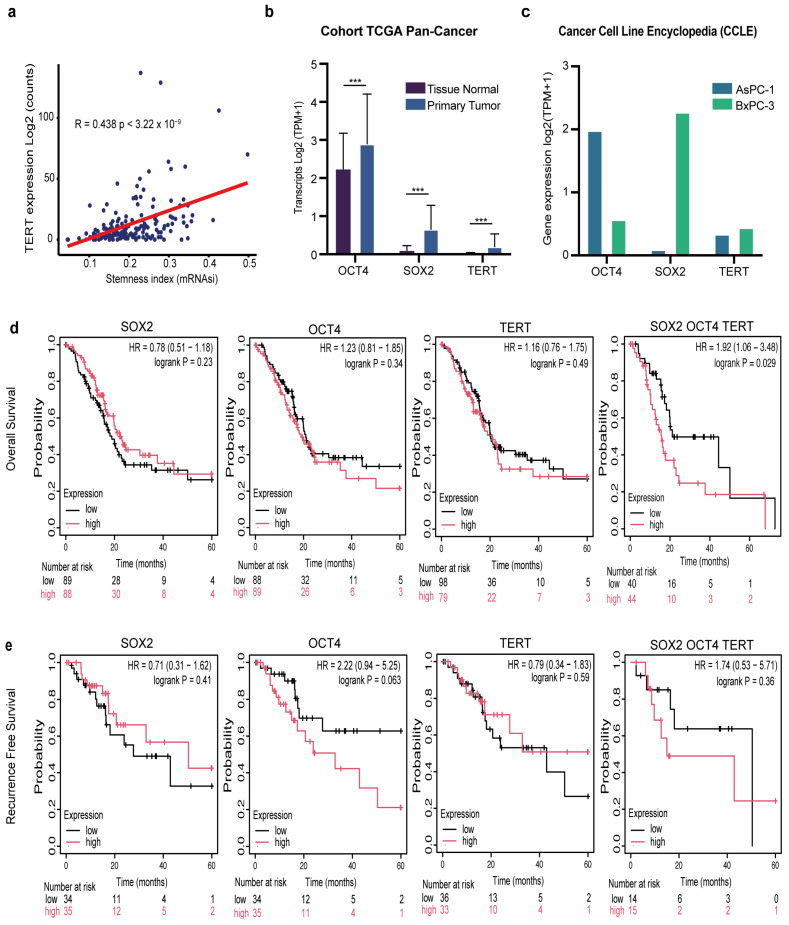
Clinical and transcriptomic relevance of *SOX2*, OCT4, and *TERT* in pancreatic cancer. (**a**) Correlation between *TERT* expression (log_2_ counts) and stemness index (mRNAsi) across pancreatic tumors (Pearson’s r = 0.438, *p* = 3.22 × 10^−9^). (**b**) Differential expression of *SOX2*, OCT4, and *TERT* in the TCGA pan-cancer cohort comparing primary tumors with matched normal tissues (log_2_ TPM + 1). Data are presented as the mean ± SD *** *p* < 0.001 Student’s *t*-test. (**c**) Expression of *SOX2*, OCT4, and *TERT* in pancreatic cancer cell lines AsPC-1 and BxPC-3 from the CCLE database (log_2_ TPM + 1). (**d**) Kaplan–Meier overall survival (OS) analysis of patients with PDAC stratified by low (black) versus high (red) expression of *SOX2*, OCT4, *TERT*, or the combined *SOX2*/OCT4/*TERT* signature. Hazard ratios (HR), 95% confidence intervals (CI), and log-rank *p*-values are shown. (**e**) Kaplan–Meier recurrence-free survival (RFS) analysis for the same gene sets as in (**d**). Hazard ratios (HR), 95% confidence intervals (CIs), log-rank *p*-values, and numbers at risk are shown in each panel.

Cancer cells exhibit stem-like characteristics, and active telomerase is a feature of stem cells [19,22]. Therefore, we examined the association between pancreatic cancer stemness and telomerase activity. To address this, we leveraged a pan-cancer stemness index derived from a transcriptional signature of 12,945 genes, originally defined by comparing embryonic stem cells with their differentiated progeny. In the pancreatic cancer cohort, we observed a significant positive correlation between stemness scores and *TERT* mRNA expression (R = 0.43, *p* = 3.2 × 10^−9^), indicating that tumors with higher telomerase levels exhibit stronger stem-like transcriptional programs (Figure 1a).

Having established the link between telomerase and pancreatic cancer stemness, and because *SOX2* and OCT4 are established master regulators of stemness [19], we next analyzed the expression of the canonical pluripotency factors *SOX2* and OCT4, together with *TERT*, in both patient-derived tumors and experimental models. Analysis of TCGA pancreatic cancer samples revealed a significant (*** *p* < 0.001) upregulation of *SOX2*, OCT4, and *TERT* in primary tumors compared to normal tissue (Figure 1b). Importantly, this pattern was recapitulated in established pancreatic cancer cell lines. Both AsPC-1 and BxPC-3 cells showed robust expression of these three genes (Figure 1c). This confirms that their overexpression is not restricted to clinical samples but represents a reproducible hallmark across experimental systems.

Several studies have shown that TERT and cancer stemness are associated with poor prognosis in patients with pancreatic cancer [23,24]. We also examined the prognostic relevance of *SOX2*, OCT4, and *TERT* expression in patients with pancreatic cancer. Kaplan–Meier analysis of the TCGA cohort demonstrated that, when considered individually, high expression of *SOX2* (*p* = 0.23), OCT4 (*p* = 0.34), or *TERT* (*p* = 0.49) did not significantly impact overall survival (OS) (Figure 1d). Strikingly, however, patients whose tumors co-expressed high levels of all three genes displayed significantly reduced OS compared with their low-expression counterparts (*p* = 0.029) (Figure 1d). A similar trend was observed when recurrence-free survival (RFS) was analyzed. High *SOX2* or *TERT* expression alone was not associated with a shorter RFS. However, patients with elevated OCT4 expression exhibited a strong tendency toward early recurrence, although this did not reach statistical significance (HR = 2.21, *p* = 0.056) (Figure 1e). Notably, combined overexpression of *SOX2*/OCT4/*TERT* also predicted poorer RFS, but this was not significant (Figure 1e). Together, these findings reveal that high co-expression of *SOX2*, OCT4, and *TERT* predicts worse overall survival, with a non-significant trend toward shorter recurrence-free survival and may be associated with another biological process.

### 3.2. Identification of a Minority Population SOX2^+^OCT4^+^TERT^high^ of Pancreatic Tumor Cell Population

**Figure 2:**
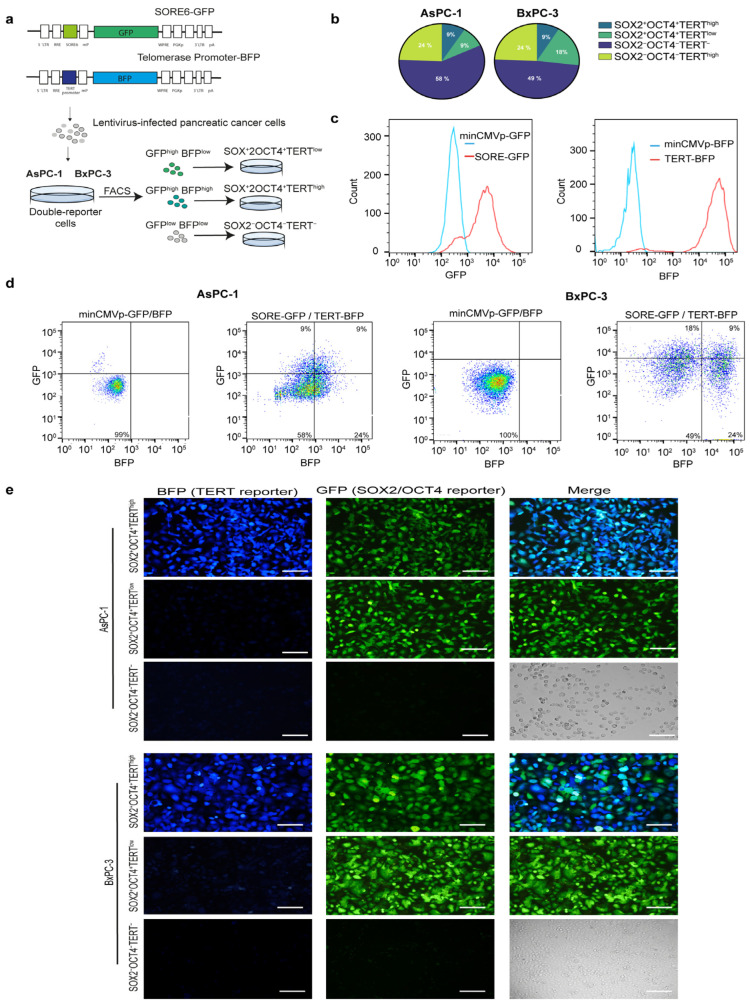
Generation and validation of dual reporter pancreatic cancer cell lines to identify SOX2^+^OCT4^+^TERT^high^ subpopulations. (**a**) Schematic representation of lentiviral reporter constructs. TERT^high^ cells were identified using a vector in which the expression of BFP was controlled by the TERT gene promoter. SOX2^+^OCT4^+^ cells were identified using the SORE6 reporter, in which the *SOX2*/OCT4 composite response element drives the expression of GFP. Pancreatic cancer cells infected with lentiviruses were subjected to FACS and puromycin selection. GFP^+^BFP^high^, GFP^+^BFP^low^, and GFP^−^BFP^−^ pancreatic cancer cells were sorted using FACS. (**b**) Pie charts showing the distribution of reporter-positive populations in AsPC-1 and BxPC-3 cells following double-reporter transduction, quantified from the representative flow cytometry plots shown in (**c**,**d**). (**c**,**d**) Representative flow cytometry plots illustrating GFP and BFP expression in AsPC-1 and BxPC-3 cells. A minimal CMV control vector lacking SORE6 or TERT promoter elements was used as a gating control. (**e**) Representative fluorescence microscopy images of sorted SOX2^+^OCT4^+^TERT^high^, SOX2^+^OCT4^+^TERT^low^, and SOX2^−^OCT4^−^TERT^−^ subpopulations in AsPC-1 and BxPC-3 cell lines. The Green-, Blue-Fluorescent Protein and merged channels are shown. Scale bars = 100 μm.

**Figure A1:**
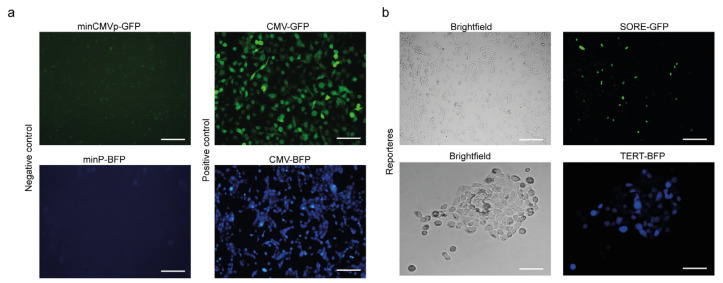
Validation of stemness (SORE-GFP) and telomerase (TERT-BFP) reporter activities in cultured cells. The minimal promoter (minCMVp-GFP, minP-BFP; negative control) yielded no detectable fluorescence, whereas the constitutive CMV promoter (CMV-GFP/BFP; positive control) showed robust green or blue expression, respectively (**a**). SORE-GFP (*SOX2*/OCT4 response element) labels a CSCs subpopulation, and TERT-BFP (*TERT* promoter) labels cells with telomerase transcriptional activity. Brightfield images of each condition were used to show the cell morphology and density. Representative live-cell fluorescence micrographs (**b**). Scale bars = 100 μm.

Next, we investigated whether TERT is expressed in pancreatic CSCs and the functional contribution of TERT-expressing tumor cells and TERT-negative cells. To identify a telomerase-positive cancer stem-like subpopulation within pancreatic cancer cell lines, we established a dual lentiviral reporter strategy to simultaneously monitor *SOX2*/OCT4 and TERT activity. For telomerase, we engineered a lentiviral reporter construct in which the human TERT promoter was coupled to a minimal promoter driving BFP expression, enabling the identification of TERT cells. To label pancreatic CSCs SOX2^+^OCT^+^, we employed the SORE6 reporter, a previously validated system that identifies the CSCs population [20]. The SORE6 reporter contains six tandem repeats of a composite *SOX2*/OCT4 response element driving GFP expression. Thus, cells co-expressing *SOX2*/OCT4 and TERT could be visualized through dual GFP and BFP positivity (Figure 2a). To generate double-reporter cell lines, human AsPC-1 and BxPC-3 pancreatic tumor cells were genetically modified by transduction with lentiviral TERT-BFP and SORE6-GFP vectors for stable expression (Figure 2a). Validation of both reporters is shown in Appendix A: minimal promoter constructs yielded no detectable signals, whereas CMV controls were robustly fluorescent (Figure A1 Appendix A). Flow cytometry analyses revealed that dual GFP and BFP positivity (GFP^+^BFP^high^) defined a SOX2^+^OCT4^+^TERT^high^ minority population, representing approximately 9% of cultured cells in both AsPC-1 and BxPC-3 cells. A comparable frequency of GFP^+^BFP^low^ (SOX2^+^OCT4^+^TERT^low^) cells, ranging from 9–18% depending on the cell line, was detected, indicating that reporter activation was restricted to a subset of tumor cells. Importantly, approximately 50% of the cells were telomerase-positive but negative for the stemness reporter (GFP^−^BFP^+^). Cells transduced with minimal promoter constructs lacking either the SORE6 element or TERT promoter served as gating controls and did not exhibit reporter activity (Figure 2b–d). Fluorescence microscopy further validated the reporter activity in the sorted subpopulations. SOX2^+^OCT4^+^TERT^high^ cells exhibited robust dual GFP and BFP fluorescence, whereas SOX2^+^OCT4^+^TERT^low^ and SOX2^−^OCT4^−^TERT^−^ fractions showed correspondingly reduced or absent reporter expression (Figure 2e). Collectively, our data revealed an SOX2^+^OCT4^+^TERT^high^ subpopulation in AsPC-1 and BxPC-3 cell lines.

### 3.3. The Subpopulation SOX2^+^OCT4^+^TERT^high^ Is Enriched for Stem Cell Transcription Factors

**Figure 3:**
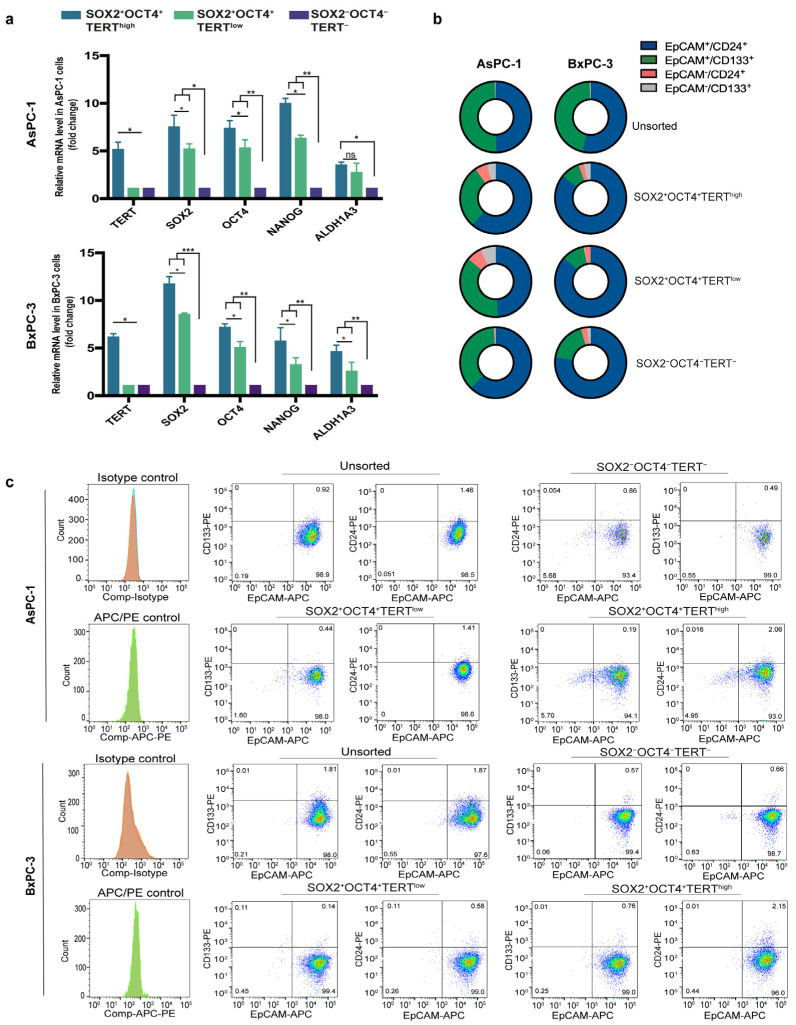
Stemness validation of SOX2^+^OCT4^+^TERT^high^ subpopulations and comparison with conventional CSC markers. (**a**) Relative mRNA expression of *SOX2*, OCT4, *TERT*, *NANOG*, and *ALDH1A1* in FACS-sorted SOX2^+^OCT4^+^TERT^high^, SOX2^+^OCT4^+^TERT^low^, and SOX2^−^OCT4^−^TERT^−^ subpopulations from AsPC-1 and BxPC-3 cell lines, measured by qPCR. Gene expression was normalized to PPIA, and the relative levels between samples were calculated using the 2^−ΔΔCt^ method. Data are presented as the mean ± SD; * *p* < 0.05, ** *p* < 0.01, *** *p* < 0.001; one-way ANOVA with Tukey’s post hoc test. (**b**) Schematic representation of the distribution of classical CSC markers (EpCAM^+^CD24^+^ and EpCAM^+^CD133^+^) across unsorted, SOX2^+^OCT4^+^TERT^high^, SOX2^+^OCT4^+^TERT^low^, and SOX2^−^OCT4^−^TERT^−^ fractions from AsPC-1 and BxPC-3 cells. (**c**) Representative flow cytometry plots showing the experimental data underlying the distributions summarized in panel B. The dot plots are displayed using a pseudocolor (density) scale to visualize the local density of events: blue indicates low event density, whereas green/yellow/red indicate progressively higher event density.

To validate our reporter-defined subpopulations at the expression level, we analyzed the mRNA expression levels of *SOX2*, OCT4, and *TERT*. In addition, we measured *NANOG* and *ALDH1A1*, two canonical stemness markers, in the FACS-sorted fractions. Using qPCR, we found that the SOX2^+^OCT4^+^TERT^high^ population preferentially expressed CSC-associated markers compared to the negative fraction (Figure 3a). Although SOX2^+^OCT4^+^TERT^low^ cells also showed increased expression of these markers, their levels remained consistently lower than those of the TERT^high^ fraction (Figure 3a).

Moreover, because pancreatic CSCs have also been defined by a combination of cell surface markers, most commonly CD44^+^CD24^+^EpCAM^+^ [9] and CD133^+^ [6], we next asked whether this reporter-defined stem-like subpopulation corresponded to these conventional markers. Surprisingly, flow cytometry analyses revealed that although EpCAM^+^CD24^+^ and EpCAM^+^CD133^+^ populations are present, their proportions do not show an increase in SOX2^+^OCT4^+^TERT^high^ or SOX2^+^OCT4^+^TERT^low^ (Figure 3b,c).

Together, these findings provide transcriptional validation of the reporter-defined populations and indicate that the SOX2^+^OCT4^+^TERT^high^ population represents a stem-like subset that is molecularly distinct from those identified by conventional surface markers. This observation supports the notion that pancreatic CSCs are heterogeneous and that different identification strategies may capture partially overlapping but biologically distinct subpopulations of cells.

### 3.4. The Subpopulation SOX2^+^OCT4^+^TERT^high^ Exhibits Elevated Proliferative, Migratory and Invasive Capacities

**Figure 4:**
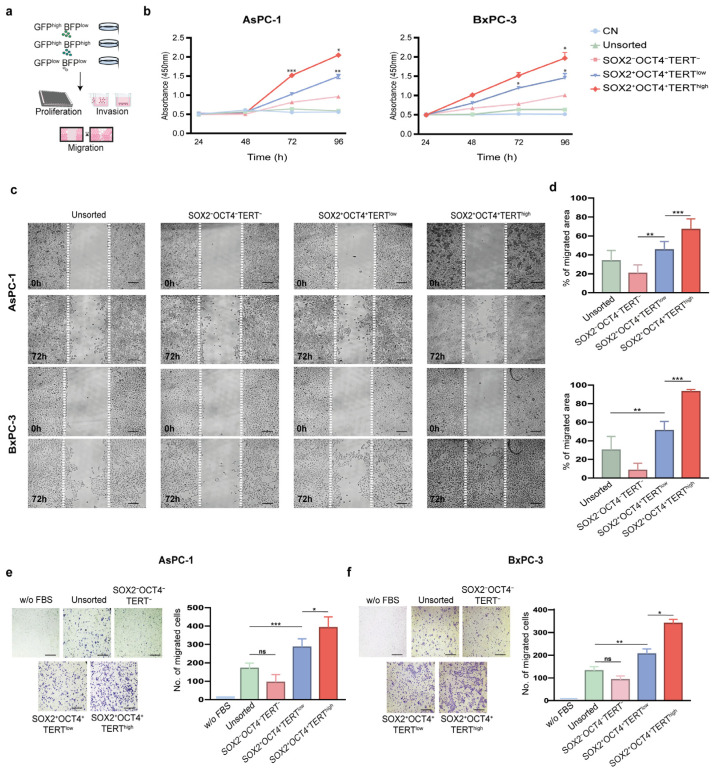
Functional characterization of SOX2^+^OCT4^+^TERT^high^ subpopulations in pancreatic cancer cell lines. (**a**) Experimental workflow: FACS isolation of subpopulations from the dual-reporter AsPC-1 and BxPC-3 lines, followed by proliferation, migration, and invasion assays. (**b**) Cell proliferation assessed by MTS assay in sorted subpopulations (SOX2^+^OCT4^+^TERT^high^, SOX2^+^OCT4^+^TERT^low^, SOX2^−^OCT4^−^TERT^−^) and unsorted AsPC-1 and BxPC-3 cells. Ara-C-treated cells were used as negative controls (CN). Wound-healing assay in AsPC-1 and BxPC-3 cells. Representative images at 0 h and 72 h (**c**) and quantification of wound closure (**d**) are presented. Invasion assays in AsPC-1 (**e**) and BxPC-3 (**f**) subpopulations. Representative images and quantification of the invading cells are shown. Medium without FBS was used as a negative control. (**d**,**e**) Data are presented as the mean ± SD; * *p*< 0.05, ** *p* < 0.01, *** *p* < 0.001; one- or two-way ANOVA with Tukey’s post hoc test. Scale bars = 50 μm.

Telomerase is vital for the ability of malignant cells to replicate, while invasion and migration are key characteristics of cancer stem cells [6,25]. To test whether reporter-defined subpopulations exhibited functional differences, we compared the growth and motility of SOX2^+^OCT4^+^TERT^high^ and SOX2^+^OCT4^+^TERT^low^ AsPC-1 and BxPC-3 cells (Figure 4a). We first assessed the proliferative capacity using the MTS assay. SOX2^+^OCT4^+^TERT^high^ cells displayed the most pronounced proliferative activity, with significantly (*p* < 0.05) higher growth rates than SOX2^+^OCT4^+^TERT^low^ cells at 72 and 96 h (Figure 4b). Although less pronounced, SOX2^+^OCT4^+^TERT^low^ cells also maintained a measurable growth advantage over TERT^−^ and unsorted populations, which consistently exhibited the lowest proliferation rates in the study. As a negative control, there was no significant change in the proliferation of Ara-C-treated cells.

Cell migration allows cells to spread toward distant sites and colonize tissues; therefore, we determined the migration potential of SOX2^+^OCT4^+^TERT^high^ and SOX2^+^OCT4 TERT^low^ cells using wound-healing assays. SOX2^+^OCT4^+^TERT^high^ cells exhibited markedly (*p* < 0.001) accelerated wound closure at 72 h, surpassing SOX2^+^OCT4^+^TERT^low^ cells, which themselves migrated more efficiently than SOX2^−^OCT4^−^TERT^−^ and unsorted fractions (Figure 4c,d). Next, we determined the invasive potential of SOX2^+^OCT4^+^TERT^high^ and SOX2^+^OCT4^+^TERT^low^ using Matrigel-coated Boyden chambers. We employed FBS as a chemoattractant and medium without FBS as a negative control. Consistent with the migration assays, a clear hierarchy was observed: SOX2^+^OCT4^+^TERT^high^ cells exhibited significantly (*p* < 0.001) higher migration abilities than SOX2^+^OCT4^+^TERT^low^ cells, which nevertheless retained higher invasive activity than the negative and unsorted populations (Figure 4e,f).

Collectively, these results establish a functional hierarchy in which SOX2^+^OCT4^+^ TERT^high^ cells represent the most aggressive subpopulation with respect to proliferation, invasion, and migration abilities. SOX2^+^OCT4^+^TERT^low^ cells also retained enhanced functional capacity relative to negative and unsorted cells, underscoring the stepwise contribution of TERT in driving aggressive cancer stem-like cell-associated phenotypes.

### 3.5. Comparative Transcriptomic Analysis of Pancreatic Cancer Subpopulation SOX2^+^OCT4^+^TERT^high^

**Figure 5:**
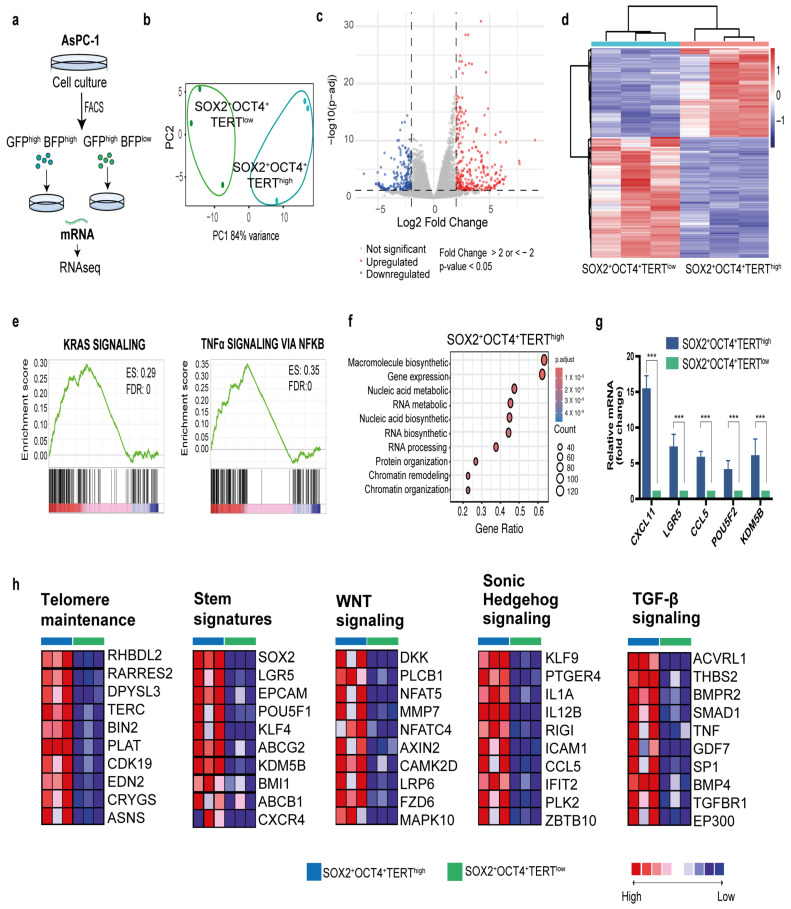
Transcriptome analysis of reporter-defined subpopulations reveals a stem-like, telomerase-driven program in PDAC. (**a**) Experimental workflow: FACS isolation of SOX2^+^OCT4^+^TERT^high^ and SOX2^+^OCT4^+^TERT^low^ cells from the dual-reporter AsPC-1 line, followed by bulk RNA-seq. (**b**) Principal component analysis of gene-expression profiles of SOX2^+^OCT4^+^TERT^high^ and SOX2^+^OCT4^+^TERT^low^ samples (**c**) The volcano plot showing the differential expression genes (DEGs) between SOX2^+^OCT4^+^TERT^high^ and SOX2^+^OCT4^+^TERT^low^. Fold change >2 or <−2, *p*-value < 0.05, and FDR < 0.05. Red and blue dots represent upregulated and downregulated genes, respectively. (**d**) Heatmap representation of differentially expressed gene grouping samples by SOX2^+^OCT4^+^TERT^high^ and SOX2^+^OCT4TERT^low^. (**e**) Gene set enrichment analysis (GSEA) comparing SOX2^+^OCT4^+^TERT^high^ and SOX2^+^OCT4^+^TERT^low^, the enriched gene sets and Enrichment Score (ES) are shown. (**f**) Gene Ontology analysis revealing biological processes for genes upregulated in TERT^high^. (**g**) Quantification of gene expression by qPCR showing the validation of representative transcripts comparing TERT^high^ and TERT^low^. Gene expression was normalized to PPIA, and the relative level between samples was calculated using the 2^−ΔΔCt^ method. Data are presented as the mean ± SD; *** *p* < 0.001; one-way ANOVA with Tukey’s post hoc test. (**h**) GSEA corresponding heatmaps associated with telomere maintenance components, stemness signatures, and pathways linked to CSC biology and invasiveness, WNT, Hedgehog, TGF-β, and EMT. Red, pink, light blue, and dark blue denote high, moderate, low, and lowest expression values, respectively. The complete pathway-focused heatmaps are provided in the Figure A2 Appendix A.

**Figure A2:**
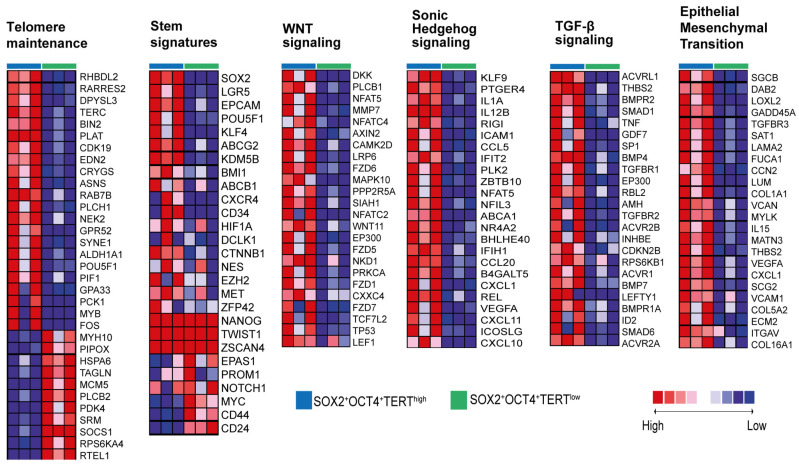
Pathway-focused heatmaps of representative gene programs enriched in SOX2^+^OCT4^+^TERT^high^ cells. Heatmaps display scaled expression of selected gene sets related to telomere maintenance, stemness signatures, WNT signaling, Sonic Hedgehog signaling, TGF-β signaling, and epithelial–mesenchymal transition (EMT) in FACS-sorted SOX2^+^OCT4^+^TERT^high^ versus SOX2^+^OCT4^+^TERT^low^ fractions. Red and blue indicate higher and lower relative expression, respectively. Related with Figure 5h.

We identified a subpopulation of pancreatic cancer cells characterized by SOX2^+^OCT4^+^TERT^high^ that exhibited increased expression of CSC markers and superior proliferative, invasive, and migratory capacities. To gain molecular insight, we performed bulk RNA-seq on FACS-sorted SOX2^+^OCT4^+^TERT^high^ and SOX2^+^OCT4^+^TERT^low^ subpopulations from the AsPC-1 line (Figure 5a). Principal component analysis (PCA) of transcriptomes enabled the clear classification of these two subpopulations, suggesting different molecular profiles (Figure 5b). Indeed, analysis of differentially expressed genes using DESeq2 revealed that 276 genes were upregulated in SOX2^+^OCT4^+^TER^high^ cells and 203 genes were downregulated (fold change > 2 or <−2, *p*-value < 0.05, and FDR < 0.05) (Figure 5c,d). Pathway-level interrogation corroborated a proliferative and transcriptionally active phenotype in TERT^high^ cells. Gene set enrichment analyses showed significant positive enrichment of KRAS signalling and TNF–NFκB signalling (FDR < 0.001), consistent with oncogenic programmes linked to PDAC aggressiveness (Figure 5e). Concordantly, Gene Ontology enrichment of upregulated genes highlighted categories related to macromolecule and nucleic acid metabolism, RNA biosynthesis/processing, ribosome biogenesis, chromatin organization/remodeling, and gene expression (Figure 5f), collectively indicating an active growth and transcriptional state. Next, we focused on genes enriched in stem cells to understand the transcriptional programs that may functionally maintain the stem cell state. At the module level, curated gene-set heatmaps demonstrated coordinated upregulation of TERT^high^ telomere-maintenance components, multiple stemness signatures, and signalling axes frequently implicated in cancer stem cell biology—WNT, Hedgehog, and TGF-β—together with a robust EMT program (Figure 5h and Figure A2 Appendix A). Finally, to validate the sequencing data, we employed qPCR to quantify the expression levels of differentially expressed mRNAs (Figure 5g). Our data showed that all transcripts analyzed (*CXCL11*, *LGR5*, *CCL5*, *POU5F1*, and *PDGFB*) were successfully validated and exhibited higher expression (*p* < 0.001) in TERT^high^ versus TERT^low^.

Together, these transcriptomic data mechanistically explain the functional hierarchy established experimentally and support the idea that SOX2^+^OCT4^+^TERT^high^ expression is associated with a stem-like, telomerase-dependent, invasion-competent transcriptional state characterized by the activation of cell cycle/biogenesis programs, KRAS signalling, and EMT.

### 3.6. Network Analysis and Identification of Potential Therapeutic Compounds Targeting the SOX2^+^OCT4^+^TERT^high^ Subpopulation

**Figure 6:**
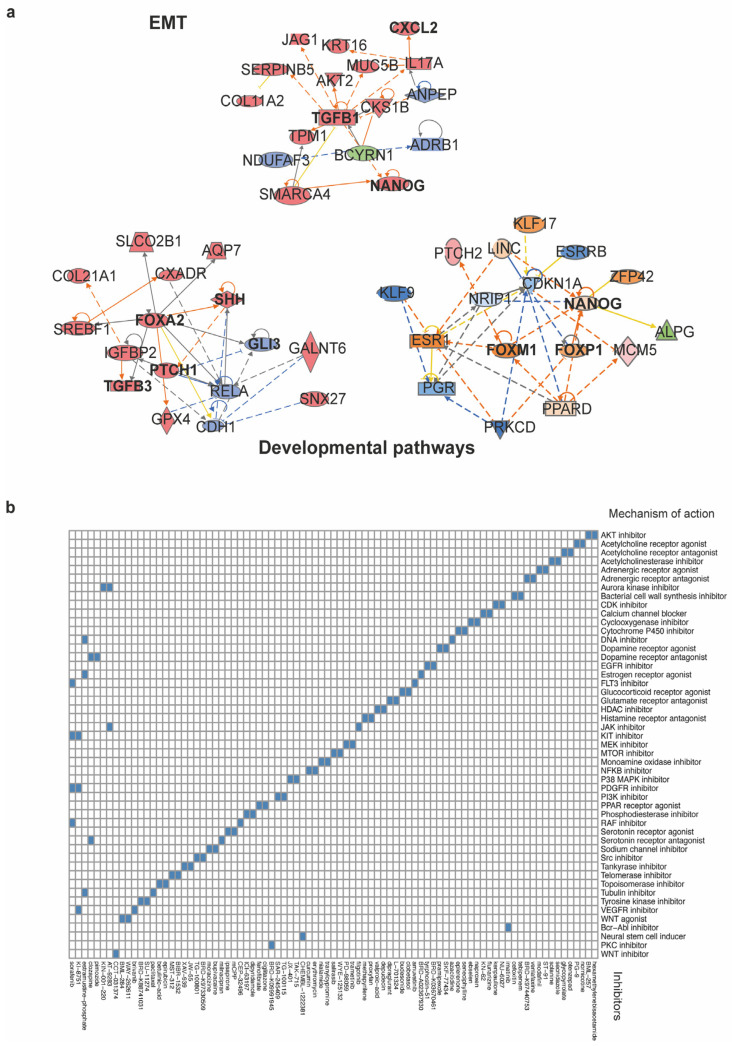
Network analysis and connectivity mapping nominate therapeutic vulnerabilities of the SOX2^+^OCT4^+^TERT^high^ subpopulation. (**a**) Top IPA networks generated from differentially expressed genes in the TERT^high^ subpopulation. The analysis revealed modules linked EMT and developmental pathways, consistent with a stem-like invasive program. Orange and blue edges indicate predicted activation or inhibition, whereas gray edges indicate relationships not predicted. Solid lines denote direct interactions, and dashed lines denote indirect interactions, as curated by IPA. The complete IPA network outputs are provided in Figure A3 Appendix A (**b**) Heatmap of compounds showing negative connectivity to the TERT^high^ transcriptional signature. Columns are perturbagens; rows are MoA classes. The full list of compounds and scoring is provided in Table S1.

**Figure A3:**
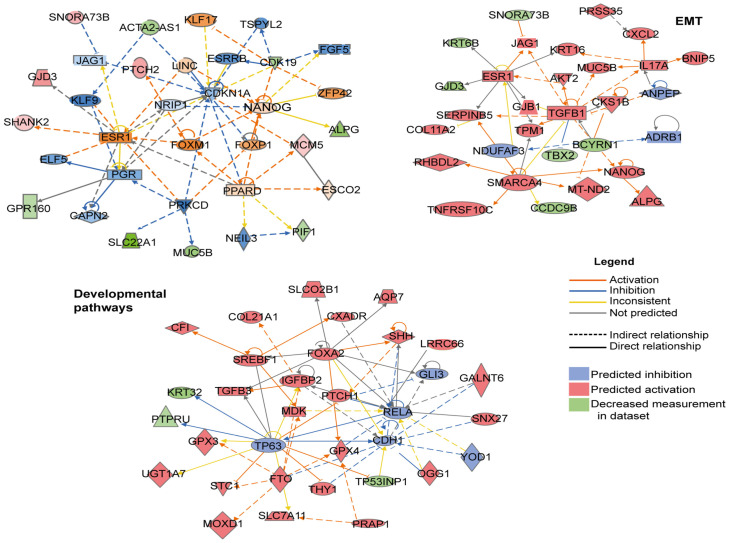
Complete IPA networks derived from the SOX2^+^OCT4^+^TERT^high^ differential expression signature. Full Ingenuity Pathway Analysis (IPA) network outputs corresponding to the EMT and developmental-pathway modules shown in Figure 6a, displayed with all nodes and interactions included by IPA.

IPA software enables the analysis of transcriptomic and other omics data within a biological context. Given that SOX2^+^OCT4^+^TERT^high^ cells exhibit enhanced aggressive properties, their differentially expressed genes were mapped onto a global molecular network curated from the Ingenuity Pathway Knowledge Base. Networks of these genes were algorithmically constructed based on their known and predicted protein-protein interrelationships. This analysis identified subnetworks built around distinct biological pathways, providing a systems-level view of the core programs that may drive pancreatic cancer growth. These programs identified the developmental and EMT pathways. The networks included genes such as TGFB1, FOXA2, PTCH, SHH, NANOG, and FOXP1 (Figure 6a and Figure A3 Appendix A).

First, a stemness module grouped pluripotency- and fate–specifying factors (e.g., NANOG, KLF5, and FOXI/FOXI1 family) and Notch ligands/receptors (JAG1). The inferred state of several of these regulators was consistent with activation, supporting the engagement of programs linked to self-renewal and lineage. The EMT network was organized around TGFB1, with connections to EMT-associated effectors and chromatin regulators (e.g., PMEPA1 and SMARCA4). Causal edges predicted an activated TGF-β axis and repression of epithelial features, a configuration concordant with the EMT enrichment observed in TERT^high^ transcriptomes and their enhanced motile phenotype. The developmental pathway networks highlighted the activation of WNT/β-catenin (CTNNB1), Hedgehog (SHH–PTCH), and additional stemness-linked regulators (FOXA2), indicating the engagement of embryonic programs that support self-renewal and lineage plasticity.

Together, these IPA-inferred causal architectures provide a mechanistic scaffold for the transcriptomic and functional phenotypes: TERT^high^ cells integrate EMT and developmental circuits, consistent with a telomerase-dependent, cancer stem-like state that underlies the heightened proliferation, migration, and invasion observed experimentally.

Finally, we employed the Connectivity Map (CMap), a data-driven, systematic approach for discovering associations among genes, chemicals, and biological conditions, to further predict potential therapeutic drugs that might target pathways associated with the SOX2^+^OCT4^+^TERT^high^ subpopulation. We found an enrichment of compounds associated with stemness, proliferation, migration, and invasion (Figure 6b). Some of the significantly enriched compounds that inhibit malignancy-related pathways include the AKT inhibitor BML-257, CDK inhibitor kenpaullone, EGFR inhibitor tyrphostin, NFKB inhibitor curcumin, telomerase inhibitor BIBR-1532, tankyrase inhibitor XAV-939, and WNT inhibitor CCT-031374. The CMap mode of action (MoA) analysis of the compounds revealed the top 49 mechanisms of action. Among these, we identified those mentioned above, as well as topoisomerase inhibitors, PI3K inhibitors, mTOR inhibitors, MAPK inhibitors, and tyrosine kinase inhibitors. Together, CMap points to actionable vulnerabilities in SOX2^+^OCT4^+^TERT^high^ cells and prioritizes testable strategies, including single-agent targeting of telomerase or WNT and rational combinations to suppress the stem-like program and its malignant behaviors.

## 4. Discussion

Our data show that telomerase is correlated with cancer stemness, and *SOX2*, OCT4, and TERT are expressed in pancreatic cancer, and their expression affects the survival outcomes of patients with pancreatic cancer is consistent with the prior pan-cancer and pluripotency literature [19,22]. We extend prior evidence by identifying and functionally characterizing a SOX2^+^OCT4^+^TERT^high^ subpopulation in PDAC. This coherence between telomerase and stemness signatures is aligned with the positive feedback loop between telomerase and stemness factors (*NANOG*, OCT4, *SOX2*, and *KLF4*), which are essential for pancreatic CSCs [26]. This confirms that telomerase participates in stem-like states beyond its canonical role in telomere maintenance in pancreatic cancer. Clinically, the literature links elements of this axis to adverse biology, suggesting that this transcriptional axis is not only biologically meaningful but also clinically relevant in pancreatic cancer. Telomerase activity and telomere-related gene programs have prognostic relevance in PDAC, and experiments have linked *SOX2*/OCT4 to therapy resistance and aggressive features [11,27,28]. Notably, our observation that combined high expression of *SOX2*/OCT4/*TERT*—rather than any single gene alone—associates with significantly reduced overall survival suggests cooperativity among pluripotency and telomerase programs, which is more prognostically informative than single-gene readouts.

By deploying dual transcriptional reporters for pluripotency (SORE6) and telomerase (*TERT* promoter), we uncovered a minority of PDAC cells with concurrent *SOX2*/OCT4/*TERT* activity in the tumor microenvironment. We observed the subpopulations SOX2^+^OCT4^+^TERT^high^ and SOX2^+^OCT4TERT^low^ across pancreatic cancer cell lines, which fits with the long-standing view that CSCs are heterogeneous rather than uniformly distributed. *SOX2*, OCT4, and *TERT* are overexpressed in a variety of cancers, including breast [29,30], prostate [31,32], lung [33], colorectal [34,35], and glioblastoma [36] and are associated with CSC subpopulations in these tumors. The SORE6 (*SOX2*/OCT4) system and *TERT*-promoter reporter have been tested independently in breast, prostate, and gastric cancers, and cells marked by these reporters have the expected properties of self-renewal, generation of heterogeneous offspring, high tumor- and metastasis-initiating activity, and resistance to chemotherapeutics [20,30,32,37,38]. However, approaches for detecting and targeting cancer stem-like cells expressing TERT^high^ (*SOX2*/OCT4/*TERT*) remain limited. The ability to separate these subpopulations of tumor cells should allow molecular characterization, elucidation of their molecular pathways, and explain several clinical observations in pancreatic cancer patients.

We also found that the SOX2^+^OCT4^+^TERT^high^ and SOX2^+^OCT4^+^TERT^low^ populations partially overlapped with the CSC markers previously reported (e.g., CD44/CD24/EpCAM, CD133) [6,9]. Notably, these discrepancies might be explained by the concept that there is heterogeneity even within stem cell populations, and CSCs are not stable but rather a highly plastic cell population. For example, similar to our results, reporter-positive (SORE6^+^) breast cancer cells did not express classical surface markers (CD44^+^CD24^−^, ALDH). Likewise, the discrepancies observed for CD133 expression may be related to the use of primary cells in other studies versus the cell lines used in our study. Discriminating whether reporter-positive cells represent a distinct CSC hierarchy versus a reversible transcriptional state will require single-cell genomics and imaging technologies to measure SOX2^+^OCT4^+^TERT^high^ cell states across stages.

Unlimited proliferation, together with migration and invasion, is the “hallmark of cancer”, as indicated by Hanahan and Weinberg. These capabilities are crucial for acquiring malignant cell states. Here, we provide evidence that the SOX2^+^OCT4^+^TERT^high^ subpopulation exhibits elevated proliferative, migratory, and invasive capacities, supporting the interpretation that SOX2^+^OCT4^+^TERT^high^ cells exhibit an aggressive phenotype and are not simply a latent ‘reservoir’ of stem-like cells, but instead can drive PDAC progression. Consistent with this view, previous studies have demonstrated that pancreatic CSCs marked by CD133^+^ CXCR4^+^, CD24^+^, CD44^+^ or OCT4^+^ NANOG^+^ have greater metastatic potential [6,11,39]. Telomerase is essential for telomere maintenance and also functions as a transcriptional cofactor that amplifies Wnt/β-catenin signaling and the TGF-β pathway, cooperating with NF-κB to directly regulate genes that converge on networks promoting EMT [14,40,41,42]. This observation suggests that telomerase can support proliferation and invasive competence when *TERT* and pluripotency circuits coincide.

We develop a comprehensive molecular map of pancreatic cancer stem-like cells by integrating their transcriptomics. Thus, this dataset provides a novel resource for understanding aggressive cell states and discovering new vulnerabilities in pancreatic cancer, leading to more effective therapies. At the gene and module levels, our data reinforce a state that contributes to malignant progression and stemness in SOX2^+^OCT4^+^TERT^high^ cells: enrichment of Kras and TNF-α signalling pathways is consistent with previous reports that inflammation and Kras-mutation define a cell population by chromatin accessibility patterns that drive their progression toward pancreatic neoplastic lesions [43,44]. Additionally, telomere maintenance was enriched, suggesting that telomerase is not only a marker of tumor proliferation but also a marker of pancreatic tumor stemness. In contrast, we identified the upregulation of *SOX2*, OCT4, *NANOG*, and other stemness genes, as well as developmental signalling (WNT, Hedgehog, TGF-β), along with a robust EMT signature. These results support previous findings that stemness features are associated with oncogenesis [19,45].

Using our gene expression signatures, we queried the CMap. Despite the dataset being based on a limited number of treated cell lines, the analysis selected drugs that have been shown to affect cancer stem cells with specificity. In particular, BML-257 (Akti-1/2; an AKT inhibitor) suppresses stem-like traits in glioma models, reducing sphere formation and proliferation, consistent with anti-GMT activity [46]. Tyrphostin AG1478 (EGFR inhibitor). Lower sphere formation and self-renewal in prostate and glioma CSC models [47]. BIBR-1532 (telomerase inhibitor) reduces sphere metrics, migration, EMT signatures, and telomerase, thereby supporting stemness across tumors [26]. XAV-939 (tankyrase). Destabilizes β-catenin, limiting stemness, neurospheres, and migration—directly targeting a core EMT/GMT driver [48]. CCT-031374 (β-catenin/TCF antagonist). Downstream Wnt blockade diminishes stem-like fractions, spheres, and clonogenicity [49].

These translational analyses may ultimately pave the way for the implementation of therapies for pancreatic tumors. Our results motivate rational combination therapies. Given the network architecture we observe, a plausible near-term strategy is to test TERT inhibition (pharmacologic or genetic) together with pathway co-blockade aligned with the dominant dependencies of the TERT^high^ transcriptome, for example, Wnt/β-catenin (porcupine or tankyrase inhibitors) or NF-κB (IKK inhibitors).

From a translational perspective, telomerase represents a potential vulnerability in PDAC because it is reactivated in malignant cells and supports replicative immortality, while also intersecting with stemness programs that our data link to aggressive phenotypes. Preclinical work with the oligonucleotide telomerase inhibitor imetelstat (GRN163L) has shown activity across multiple pancreatic cancer cell lines [50], supporting the feasibility of pharmacologically engaging telomerase in this disease. An additional consideration is that telomerase-directed therapies may not require continuous dosing: in pancreatic cancer models, telomerase inhibition can persist for weeks after drug withdrawal, suggesting that intermittent schedules might preserve on-target activity while mitigating cumulative toxicities [50]. Despite these opportunities, several constraints argue against telomerase inhibition as monotherapy in PDAC and warrant careful consideration. Although anti-telomerase cancer therapies have demonstrated encouraging research prospects, several potential concerns deserve careful consideration. Research findings suggest that the action of telomerase inhibitors on cancer cells may require a certain time frame to effectively shorten telomeres to a critical length, necessitating adequate periods of drug exposure for efficacious treatment, which may be incompatible with the pace of progression in advanced disease [51]. Although telomerase activity is typically low in most somatic tissues, potential on-target effects in telomerase-dependent compartments, including normal stem/progenitor pools, remain a concern, underscoring the importance of therapeutic windows, schedule optimization, and careful toxicity monitoring [52]. Additionally, telomeres serve as protective caps that maintain genomic stability in both normal and neoplastic cells. The suppression of telomerase activity in tumor cells results in telomere erosion, which could precipitate genomic instability within these malignantly transformed cells. This instability may further contribute to their carcinogenic progression.

## 5. Limitations

Several limitations should be considered when interpreting these findings. First, while the dual-reporter strategy enables prospective identification of a SOX2^+^OCT4^+^TERT^high^ state and links this fraction to aggressive phenotypes, the absence of in vivo validation limits translational inference. Future studies should test tumor-initiating capacity and metastatic competence using xenograft models, including limiting-dilution designs and metastasis assays. Second, although TERT^high^ status is strongly associated with proliferation, migration, and invasion, we did not directly demonstrate that TERT activity is required for these phenotypes. Establishing causality will require genetic (knockdown/knockout) or pharmacologic perturbation of *TERT* specifically in sorted SOX2^+^OCT4^+^TERT^high^ cells. Third, our transcriptomic analyses rely on bulk RNA-seq, which cannot fully resolve cellular heterogeneity, state transitions, or the co-occurrence of stemness and EMT-like programs at single-cell resolution; single-cell and/or spatial profiling will be important to dissect plasticity and transcriptional dynamics more rigorously. Fourth, functional assays were performed in two PDAC models (AsPC-1 and BxPC-3), but transcriptomic profiling was conducted only in AsPC-1, anchoring pathway-level inferences to a single model; extending RNA-seq or targeted validation of key signature genes and pathways to BxPC-3 will strengthen generalizability. Fifth, the clinical support for the *SOX2*/OCT4/*TERT* co-expression signature is currently derived from publicly available transcriptomic datasets and survival analyses, and we did not validate the signature in an independent cohort or confirm co-expression at the protein level; dedicated PDAC cohorts with clinicopathologic annotation and validation will be necessary to reinforce clinical credibility. Six, the therapeutic candidates nominated by CMap should be interpreted as hypothesis-generating, as we did not perform functional validation. Proof-of-concept testing in TERT^low^ versus TERT^high^ fractions will be required to substantiate differential vulnerabilities and therapeutic implications. Finally, we did not stratify PDAC by etiologic background, such as pancreaticobiliary maljunction (PBM). Given the established association of PBM with biliary carcinogenesis and its broader links to pancreaticobiliary pathology [53] future studies leveraging PBM-enriched clinical cohorts could test whether pancreatobiliary reflux contexts preferentially harbor or promote SOX2/OCT4-positive, TERT^high^ stem-like tumor states.

## 6. Conclusions

In summary, our study defines a PDAC subpopulation characterized by *SOX2*/OCT4 activity and high *TERT* expression as an aggressive state and proposes candidate compounds as potential therapeutic options. This study substantially deepens our understanding of PDAC tumor biology and cancer stem-like cell regulation and could inform improved treatments for patients.

## Data Availability

The original contributions presented in this study are included in this article/Supplementary Materials section. Further inquiries should be directed to the corresponding authors.

## Supplementary Materials

The following supporting information can be downloaded at https://www.mdpi.com/article/10.3390/cells15020129/s1; Table S1. Output from the Connectivity Map, Related to Figure 6b.

## Abbreviations

The following abbreviations are used in this manuscript.

BFP	Blue Fluorescent Protein
CMV	Cytomegalovirus
CN	Negative Control
CSCs	Cancer Stem Cells
EMT	Epithelial-to-Mesenchymal Transition
ES	Enrichment Score
FACS	Fluorescence-Activated Cell Sorting
FBS	Fetal Bovine Serum
FDR	False Discovery Rate
GFP	Green Fluorescent Protein
GSEA	Gene set enrichment analysis
HR	Hazard Ratio
OCT4	Octamer-binding transcription factor 4
OS	Overall Survival
PBM	Pancreaticobiliary maljunction
PCA	Principal Component Analysis
PCR	Polymerase Chain Reaction
PDAC	Pancreatic Ductal Adenocarcinoma
qPCR	quantitative real-time PCR
RFS	Relapse-Free Survival
SD	Standard Deviation
SOX2	Sex-determining region Y (SRY)-Box2
TCGA	The Cancer Genome Atlas
TERT	Telomerase reverse transcriptase

## Appendix B

**Table A1:** List of vectors used.

	Vector		
TERT reporter	TERTpromoter-BFP	Reporter vector telomerase	Engineered
	mP-BFP	Negative BFP vector	Engineered, base Addgene
	CMV-BFP	Positive BFP vector	Invitrogen
SOX2/OCT4 reporter	SORE6-GFP	Reporter vector SOX2^+^/OCT4^+^	Gift from Dra. Lalage M. Wakefield, NCI, USA
	minCMV-GFP	Negative GFP vector	
	pLJM1-EGFP	Positive GFP vector	Addgene

**Table A2:** List of primers (forward and reverse) used for qRT-PCR analysis and their melting temperatures (Tm).

Gene	Forward (5′-3′)	Reverse (5′-3′)	Tm (°C)
SOX2	CCACAGTTACGCGCACATGA	AGCCGTTCATGTAGGTCTGC	60
OCT4	CTCCTGGAGGGCCAGGAATC	CCACATCGGCCTGTGTATAT	60
TERT	CCGATTGTGAACATGGACTACG	CACGCTGAACAGTGCCTTC	60
NANOG	AGGCAAACAACCCACTTCTG	TCTGCTGGAGGCTGAGGTAT	60
ALDH1A3	TGTTAGCTGATGCCGACTTG	TTCTTAGCCCGCTCAACACT	60
PPIA	ATGCTGGACCCAACACAAAT	TCTTTCACTTTGCCAAACACC	60
CXCL11	CAGTTGTTCAAGGCTTCCCC	ACTTGGGTACATTATGGAGGCTT	60
LGR5	CTGCCCCACACACTGTCA	ATGTTGTTCATACTGAGGTCTAGGT	60
CCL5	CTGCCTCCCCATATTCCTCG	TCGGGTGACAAAGACGACTG	60
POU5F2	AGACAGCCCTTCTGGAAAGC	GCTGAGGTCAGTGCCTCTTT	60
KDM5B	CGGATTGGCAGCCACCAT	TCCCAGTACTTTGCAATCTGGT	60
